# An innovative pyroptosis-related long-noncoding-RNA signature predicts the prognosis of gastric cancer *via* affecting immune cell infiltration landscape

**DOI:** 10.3389/pore.2022.1610712

**Published:** 2022-12-07

**Authors:** Siping Xiong, Long Jin, Chao Zeng, Hongmei Ma, Linying Xie, Shuguang Liu

**Affiliations:** ^1^ Department of Pathology, The Eighth Affiliated Hospital, Sun Yat-sen University, Shenzhen, Guangdong, China; ^2^ Department of Pathology, Shengli Clinical Medical College, Fujian Medical University, Fuzhou, Fujian, China

**Keywords:** prognosis, gastric cancer, pyroptosis, overall survival, lncRNA signature

## Abstract

**Background:** Gastric cancer (GC) is a worldwide popular malignant tumor. However, the survival rate of advanced GC remains low. Pyroptosis and long non-coding RNAs (lncRNAs) are important in cancer progression. Thus, we aimed to find out a pyroptosis-related lncRNAs (PRLs) signature and use it to build a practical risk model with the purpose to predict the prognosis of patients with GC.

**Methods:** Univariate Cox regression analysis was used to identify PRLs linked to GC patient’s prognosis. Subsequently, to construct a PRLs signature, the least absolute shrinkage and selection operator regression, and multivariate Cox regression analysis were used. Kaplan–Meier analysis, principal component analysis, and receiver operating characteristic curve analysis were performed to assess our novel lncRNA signature. The correlation between risk signature and clinicopathological features was also examined. Finally, the relationship of pyroptosis and immune cells were evaluated through the CIBERSORT tool and single-sample lncRNA set enrichment analysis (ssGSEA).

**Results:** A PRLs signature comprising eight lncRNAs was discerned as a self-determining predictor of prognosis. GC patients were sub-divided into high-risk and low-risk groups *via* this risk-model. Stratified analysis of different clinical factors also displayed that the PRLs signature was a good prognosis factor. According to the risk score and clinical characteristics, a nomogram was established. Moreover, the difference between the groups is significance in immune cells and immune pathways.

**Conclusion:** This study established an effective prognostic signature consist of eight PRLs in GC, and constructed an efficient nomogram model. Further, the PRLs correlated with immune cells and immune pathways.

## Introduction

Gastric cancer is a worldwide popular malignant tumor, with the incidence rate of fifth and the mortality rate of fourth globally [1, 2]. More than one million new cases diagnosed every year. Since gastric cancer is often diagnosed at advanced stages, nearly 800,000 patients died worldwide in 2020 [3]. Currently, surgery is the most effective treatment for gastric cancer. Unfortunately, gastric cancer patients in advanced stage lost the surgical opportunity and could be only treated with radiotherapy, chemotherapy or neoadjuvant therapy clinically [4]. Despite the improvement of early diagnosis and the availability of new and effective chemotherapeutic regimens in gastric cancer, the overall 5-year survival remains poor in most countries of the world. In addition, the curative effect is worse in patients of advanced gastric cancer, with the median survival time less than 1 year [5, 6]. Thus the burden of gastric cancer is still heavy in most countries. Discovering novel biomarkers to predict or improve prognosis of gastric cancer is urgent.

Long non-coding RNA (lncRNA) ranging in length from 200 nucleotides to ∼100 kilobases have been shown to participate in cellular mechanisms widely at multi-levels. In addition, increasing evidence implicates that lncRNA plays an important role in cancers [7–9], cardiovascular disease [10–12] and other pathological conditions. LncRNAs regulate the occurrence and progression of various human diseases, especially in human cancers including gastric cancer [13], lung cancer [14], liver cancer [15] and other cancers [16]. LncRNA has been recognized as one of the best candidates for potential diagnostic or prognostic biomarkers of gastric cancer [17–19].

Pyroptosis is a novel form of programmed cell death, also known as cellular inflammatory necrosis [20]. Recently, it was found that pyroptosis may play a dual-role in the mechanism of cancer development and treatment [21]. First, the normal cells are stimulated by many inflammatory factors released from thermal prolapse, result in transforming into tumor cells. Meanwhile, a new therapeutic target may come from the promotion of cancer cell pyroptosis [22, 23]. However, little literature reported the function of pyroptosis in the gastric cancer [24]. Apart from that, whether lncRNA related with pyroptosis is still unclear. In light of that, this study was attempted to find pyroptosis-related lncRNAs (PRLs) signature to predict the prognosis of gastric cancer.

The gene expression data is displayed at open databases gene expression omnibus (GEO). According to bioinformatics analysis, we could find some lncRNA signatures with prognostic value. In this study, lncRNA and pyroptosis gene expression profiles in GSE62254 of gastric cancer were analyzed with multistep re‐annotation. Fortunately, some lncRNAs expression level was found to be related with pyroptosis messenger RNA (mRNA) expression (*p* < 0.001). Moreover, the PRLs signature was established with prognostic value *via* Cox regression and LASSO models. Patients with low-risk score had better overall survival than those with high-risk score notably (Log-rank test, *p* < 0.0001). Moreover, the PRLs signature could predict the prognosis in GC independently. In addition, its robustness was also well validated in the GSE62254 internal validation set and the GSE57303 external validation set. Finally, the prognosis of gastric cancer was predicted by constructing models and nomograms. In summary, the PRLs signature shows a good effect in the prognosis evaluation of patients with GC.

## Materials and methods

### Data downloading and processing

The microarray data and corresponding clinical data used in our study were downloaded from the gene expression omnibus (https://www.ncbi.nlm.nih.gov/geo/) database. The gene expression profiles were downloaded as MINiML formatted family files. The GSE62254 dataset (https://www.ncbi.nlm.nih.gov/geo/query/acc.cgi?acc=GSE62254), including data from 300 GC sample, was served as the training set and internal validation set. The GSE57303 dataset (https://www.ncbi.nlm.nih.gov/geo/query/acc.cgi?acc=GSE57303), including data from 70 GC sample, was served as an external validation set. All of the tissue specimens were obtained from primary resection of gastric cancer without chemotherapy or radiotherapy. The histological subtype of gastric cancer was adenocarcinoma in this study. A total of 2448 lncRNA-specific probes were included on Affymetrix Human Genome U133 Plus 2.0 platform.

### Identification of pyroptosis-related lncRNA probes

A set of 33 pyroptosis-related genes (PRGs) was achieved from previous studies (Supplementary Datasheet S1). The lncRNA data and PRGs were extracted from the transcriptome profiling data of GC obtained from the GSE62254 dataset. The correlation between lncRNAs and PRGs was evaluated *via* Pearson correlation analysis. Any lncRNA was considered as pyroptosis-related lncRNAs (PRLs) when the lncRNA with an absolute correlation coefficient of >0.3 and *p* < 0.001.

### Construction of a pyroptosis-related lncRNA signature

Univariate Cox regression analysis was carried out to select significant prognostic PRL probe in the training set GSE62254 (*n* = 225), and lncRNA probes with *p* < 0.05 were subjected for further analysis. After prognostic lncRNAs were gained by probe annotation, the least absolute shrinkage and selection operator (LASSO) regression analysis was performed to further select these candidate lncRNAs *via* the “glmnet” R package. Then, few of candidate lncRNAs were contained in the multivariate Cox regression analysis with the survminer package. According to the lowest Akaike information criterion (AIC) value, the optimal lncRNA risk signature was figured out. We calculated the risk score for each patient through using the following formula:

$$RiskScore=\sum_{i=1}^{n}coefficient\left(i\right)*expression\;of\;signature\;lncRNA\mathrm{(i)}.$$

### Validation of the pyroptosis-related lncRNA signature

The internal validation set GSE62254 (*n* = 75), the entire set GSE62254 (*n* = 300), and the external validation set GSE57303 (*n* = 70) were verified with the same risk formula. Based on the median risk score of the training set GSE62254 (*n* = 225), the patients were subdivided into high-risk group and low-risk group. The survival difference between the two groups was compared with Kaplan‐Meier curves. To evaluate the predictive power of the prognostic PRL signature, receiver operating characteristic (ROC) and Cox regression analyses were used. Further, principal component analysis (PCA) was performed to investigate the distribution of the two groups.

### Correlations with clinicopathological characteristics and the establishment of a nomogram

To test the independent prognosis of the lncRNA signature, univariate and multivariate Cox regression analyses were performed. Next, the association between this signature and other clinicopathological characteristics associated with the prognosis of GC was studied. In addition, stratified analysis was also examined to test the lncRNA signature based on some clinicopathological features. A nomogram including some important clinical features was constructed to estimate GC patients’ survival probability at 1, 3, and 5 years based on the entire GSE62254 dataset *via* the “rms” R package.

### Tumor immunity analysis

The correlation between risk scores and biological functions was analyzed by single-sample gene-set enrichment analysis (ssGSEA) which could evaluate the scores of infiltrating immune cells and the activity of immune-related pathways. CIBERSORT algorithm and spearman correlation were used to assess different distributions of 22 types of tumor-infiltrating immune cells (TIICs) with variation of the risk score.

### Statistical analysis

All the statistical analyses were performed with the R (version 4.1.2, http://www.r-project.org) and R Bioconductor packages. Kaplan–Meier curve was figured to analyze the differences between two groups through the log-rank test from the survival package in survival curves. Univariate and multivariate analyses were applied with Cox proportional risk regression models. ROC analysis and the area under ROC curve (AUC) were also used. In all analyses, the statistically significant difference was identified as *p* < 0.05.

## Results

### Establishing of and validation prognostic pyroptosis-related lncRNAs signature in the training set

**FIGURE 1:**
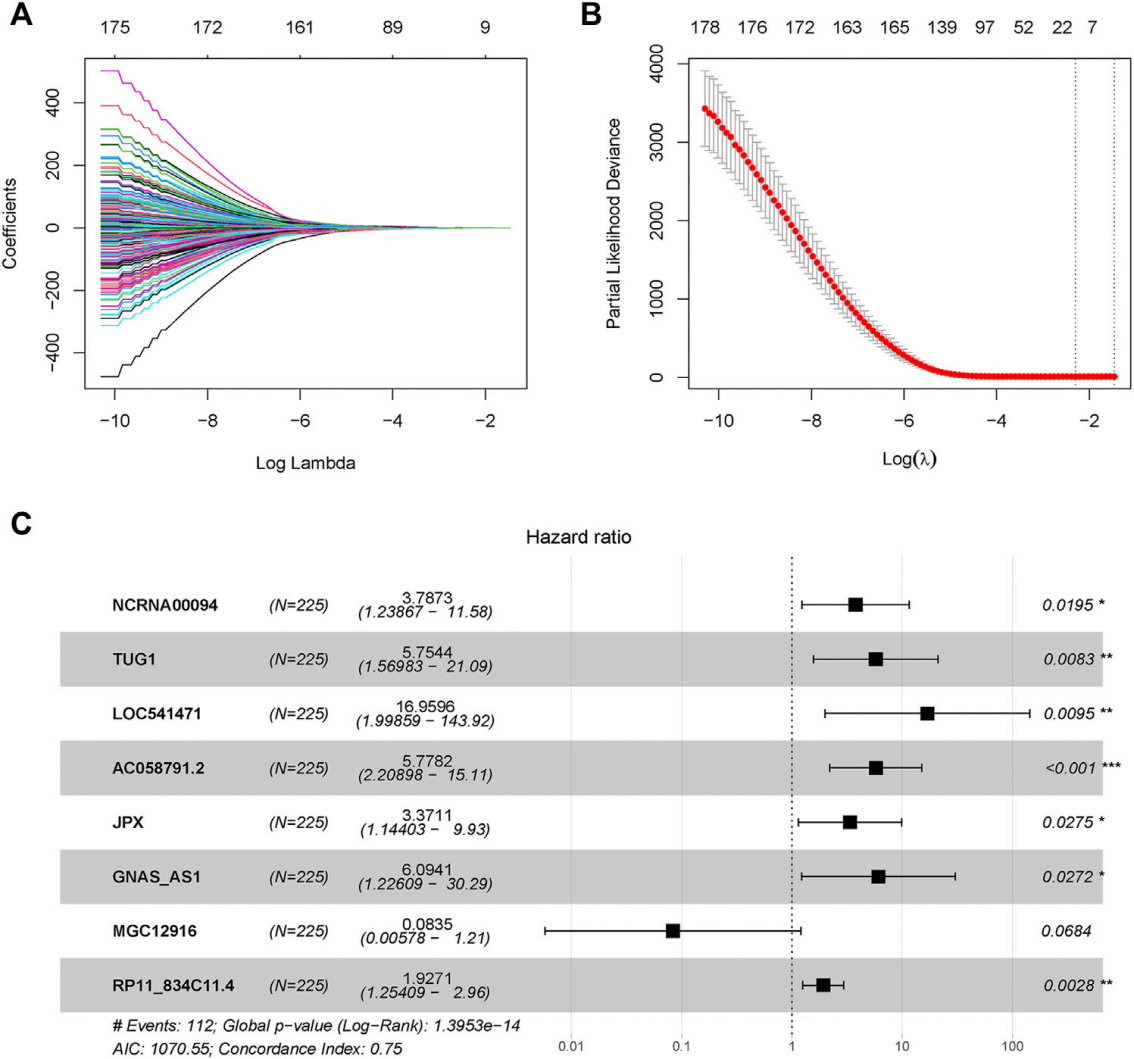
Pyroptosis-related long-noncoding-RNAs (PRLs) to predict prognosis of gastric cancer were recognized through LASSO and cox regression analysis. **(A)** LASSO coefficient spectrum of lncRNAs related with overall survival in gastric cancer. **(B)** Cross-validation error rates, each point represented a λ value accompanied with error bars which to provide a confidence interval for the cross-validated error rate. **(C)** Eight prognostic PRLs in the training set were selected *via* multivariate cox regression.

A total of 428 expressed PRL probes were identified *via* Pearson’s correlational analyses (*p* < 0.001, |Pearson correlation coefficient| > 0.3). Then, 300 GC patients were randomly sub-divided to either training set (*n* = 225) or testing set (*n* = 75) in a ratio of 3:1. In order to identify prognostic lncRNAs of GC patients, 428 PRL probes of GC were screened through univariate Cox regression analysis in the training GSE62254 set, and 230 lncRNA probes significantly associated with prognosis of GC patients were obtained (*p* < 0.05) (Supplementary Datasheet S2). After annotation of the probe, 183 annotated lncRNAs with unique symbol were retained (Supplementary Datasheet S3). To avoid over-fitting, the least absolute shrinkage and selection operator (LASSO) regression analysis was used to select candidate lncRNAs in the “glmnet” R package (Figures 1A,B). Subsequently, 16 candidate lncRNAs were preserved in the multivariate Cox regression analysis with the survminer package and the optimal lncRNA risk signature was established based on the lowest Akaike information criterion (AIC) value. Finally, an eight-lncRNA signature–based model was conatructed (Table 1 and Figure 1C). The risk score based on the signature: risk score = 1.331652× expression level of NCRNA00094 + 1.749969 × expression level of TUG1 + 2.830835 × expression level of LOC541471 + 1.754086×expression level of AC058791.2 + 1.21525 × expression level of JPX +1.807323 × expression level of GNAS-AS1-2.48323 × expression level of MGC12916 + 0.655998 × expression level of RP11-834C11.4.

**TABLE 1:** Multivariate Cox regression analysis to develop pyroptosis -based prognostic signature.

Id	Coef	HR	HR.95L	HR.95H	p Value
NCRNA00094	1.331652	3.787295	1.238673	11.57981	0.019526
TUG1	1.749969	5.754424	1.569833	21.09357	0.008281
LOC541471	2.830835	16.95962	1.998591	143.9157	0.009469
AC058791.2	1.754086	5.778166	2.208976	15.11434	0.00035
JPX	1.21525	3.371136	1.144026	9.933828	0.027525
GNAS-AS1	1.807323	6.094114	1.22609	30.28997	0.027167
MGC12916	−2.48323	0.083473	0.005778	1.205894	0.068371
RP11-834C11.4	0.655998	1.927065	1.254086	2.961185	0.002763

**FIGURE 2:**
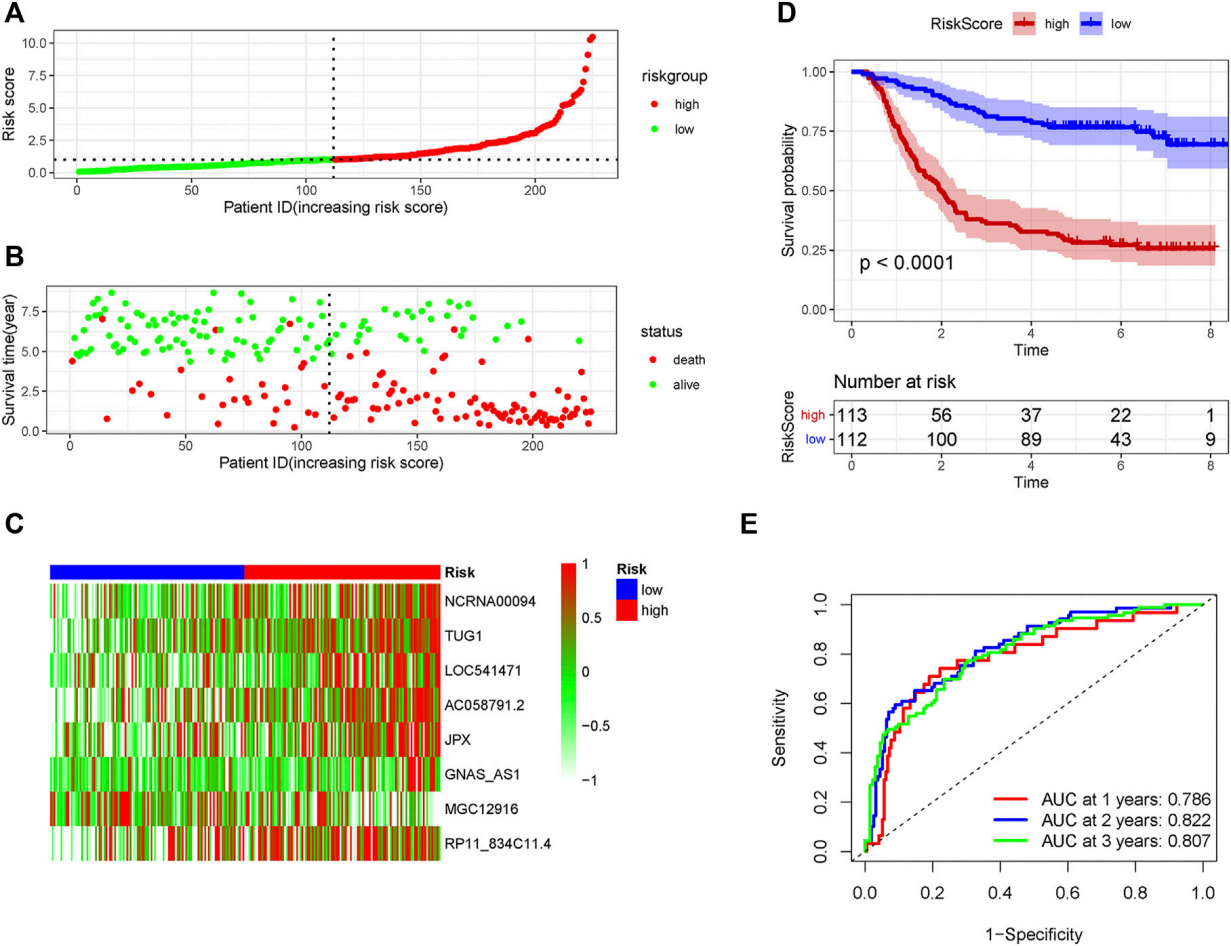
The predicting efficacy of the risk PRLs signature in the training set GSE62254 (*n* = 225). The distribution of the **(A)** risk score and **(B)** survival status. **(C)** Heatmap exhibited the lncRNAs expression level. **(D)** Kaplan-Meier survival between the two groups. **(E)** ROC curves of the cohort prediction model in 1, 2 and 3 years. For **(A)** and **(B)**, red represents dead and green represents alive; For **(C)**, red means higher expression and green means lower expression.

The patients in the GSE62254 set were subdivided into high- and low-risk group based on the median risk score. Distribution of risk scores and survival status were showed in Figure 2A and Figure 2B. The expression level of the eight lncRNAs in the high- and low-risk group was shown in Heatmap (Figure 2C). As shown in Figure 2D, patients with a high-risk score had a markedly worse survival than those with a low-risk score. In the training set, receiver operating characteristic (ROC) curve demonstrated that the AUC values for survival in 1, 2, and 3 years were 0.786, 0.822, and 0.807, respectively (Figure 2E).

### Validation of the pyroptosis-related lncRNA signature in the internal and external validation set

**FIGURE 3:**
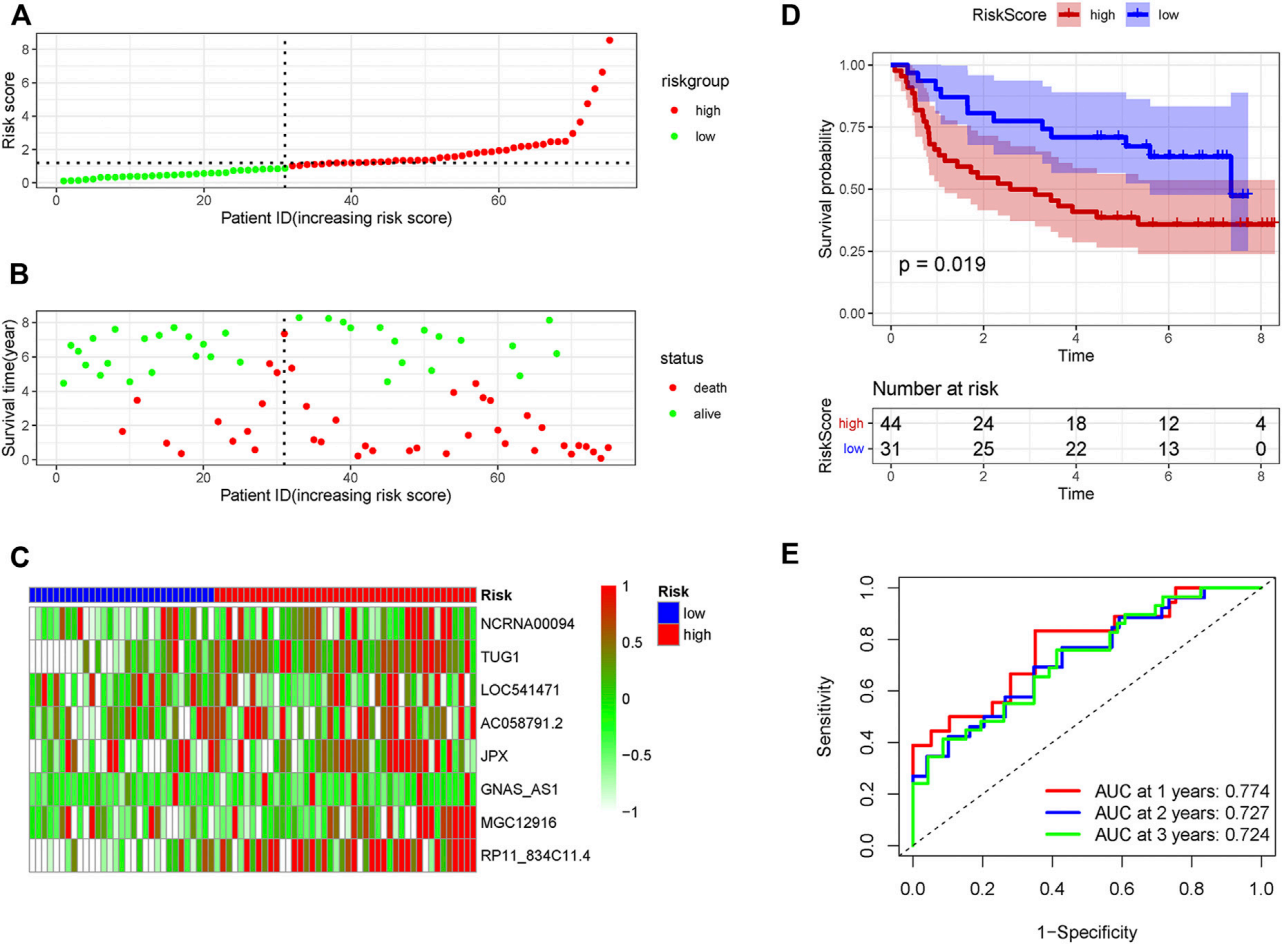
The predicting efficacy of the risk PRLs signature in internal validation set GSE62254 (*n* = 75). The distribution of the **(A)** risk score and **(B)** survival status. **(C)** Heatmap exhibited the lncRNAs expression level. **(D)** Kaplan-Meier survival between the two groups. **(E)** ROC curves of the cohort prediction model in 1, 2 and 3 years. For **(A)** and **(B)**, red represents dead and green represents alive; For **(C)**, red means higher expression and green means lower expression.

**FIGURE 4:**
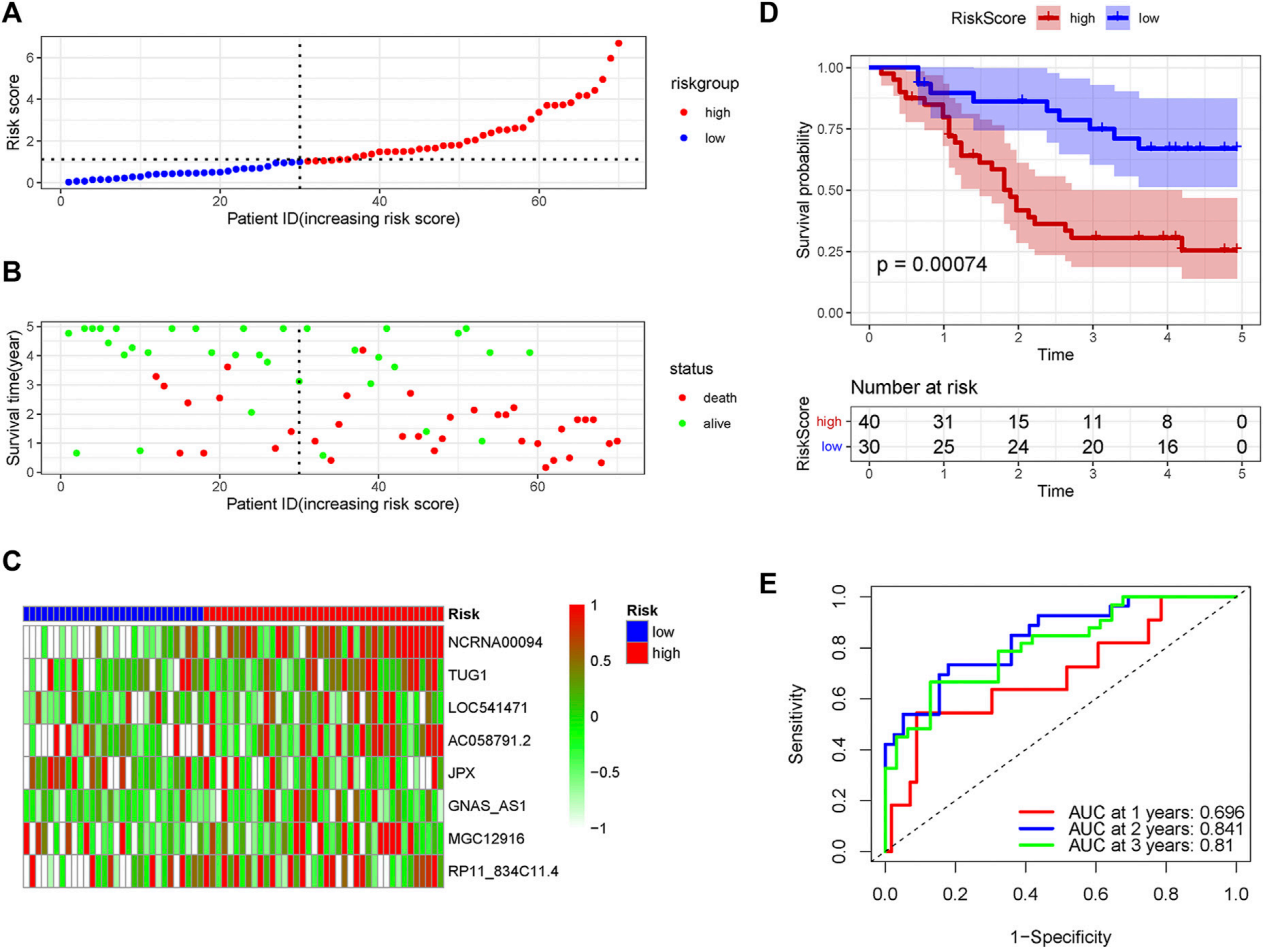
The predicting efficacy of the PRLs risk signature in external validation set GSE57303. The distribution of the **(A)** risk score and **(B)** survival status. **(C)** Heatmap exhibited the lncRNAs expression level. **(D)** Kaplan-Meier survival between the two groups. **(E)** ROC curves of the cohort prediction model in 1, 2 and 3 years. For **(A)** and **(B)**, red represents dead and green represents alive; For **(C)**, red means higher expression and green means lower expression.

**FIGURE 5:**
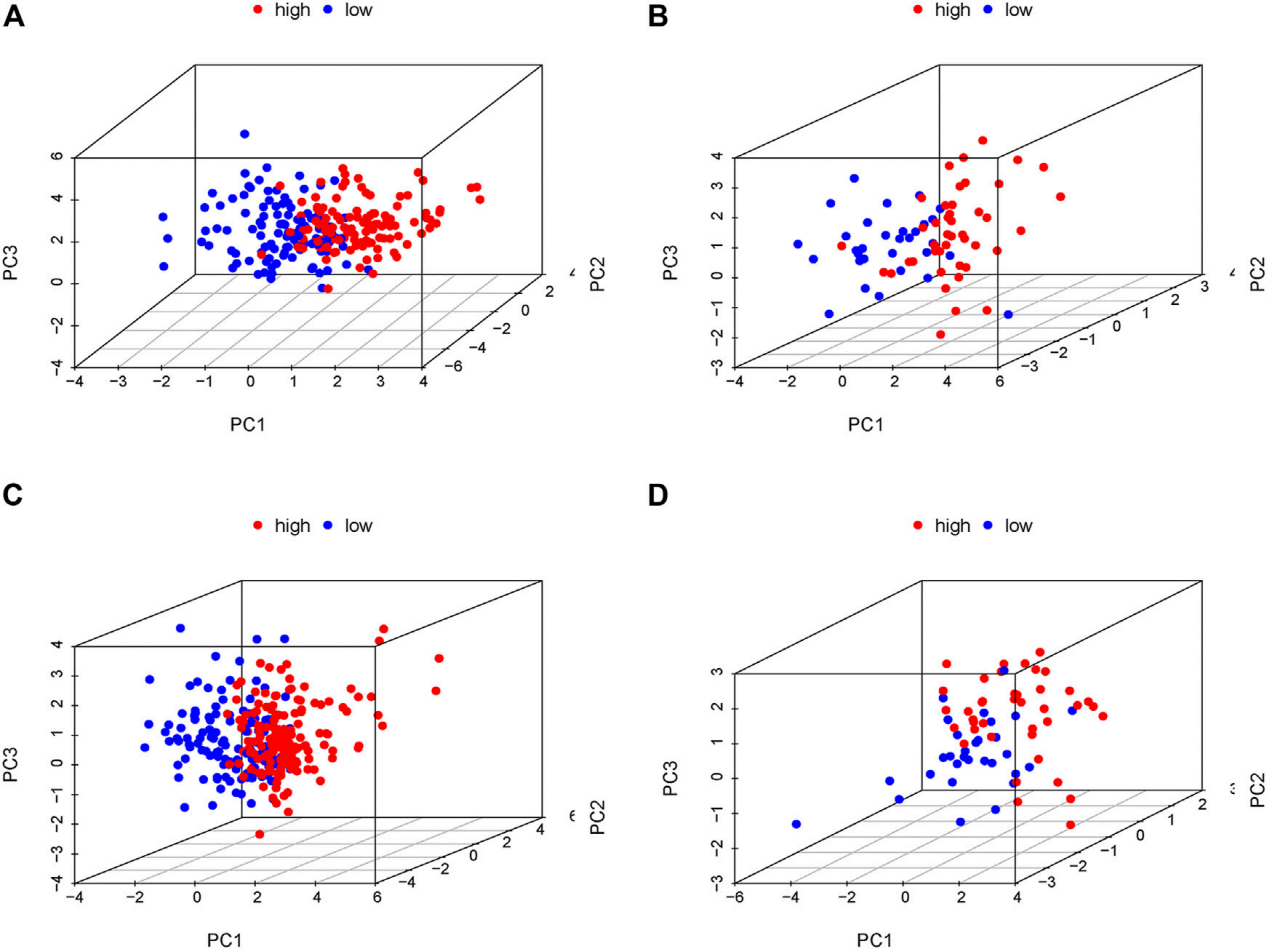
Principal component analysis (PCA) in the low- and high-risk groups according to the expression level of the eight prognostic lncRNAs. **(A)** Training cohort of GSE62254, **(B)** internal validation cohort of GSE62254, **(C)** entire cohort of GSE62254, **(D)** External validation cohort of GSE57303. Red and blue represent high-risk group and low-risk group, separately.

In order to verify the accuracy of the PRLs signature, each patient in the validation set received a risk score using the same formula as in the training set. Then, patients in the internal and external validation set were separately distributed into the low-risk group and high-risk group through using the same cutoff value as the training set. The distribution of risk scores and survival status in the two sets were displayed in Figures 3A,B, 4A,B. The expression level of eight PRLs in the high-risk and low-risk group was exhibited in heatmap (Figures 3C, 4C). In line with the training set, the GC patients in the low-risk group tended to better survival (Figures 3D, 4D). Receiver operating characteristic (ROC) curve displayed that the AUC values for survival in 1, 2, and 3 years were 0.774, 0.727, and 0.724 in the internal validation set (Figure 3E), while the values were 0.696, 0.841, and 0.81 in the external validation set (Figure 4E), respectively. Principal component analysis (PCA) revealed that GC patients in the two groups were scatted in different directions based on the expression of the eight PRLs (Figure 5). These results implied that the PRL-based prognostic features are robust and stable in prognostic prediction for GC patients.

### The clinical independence and correlation estimation of the risk signature

**FIGURE 6:**
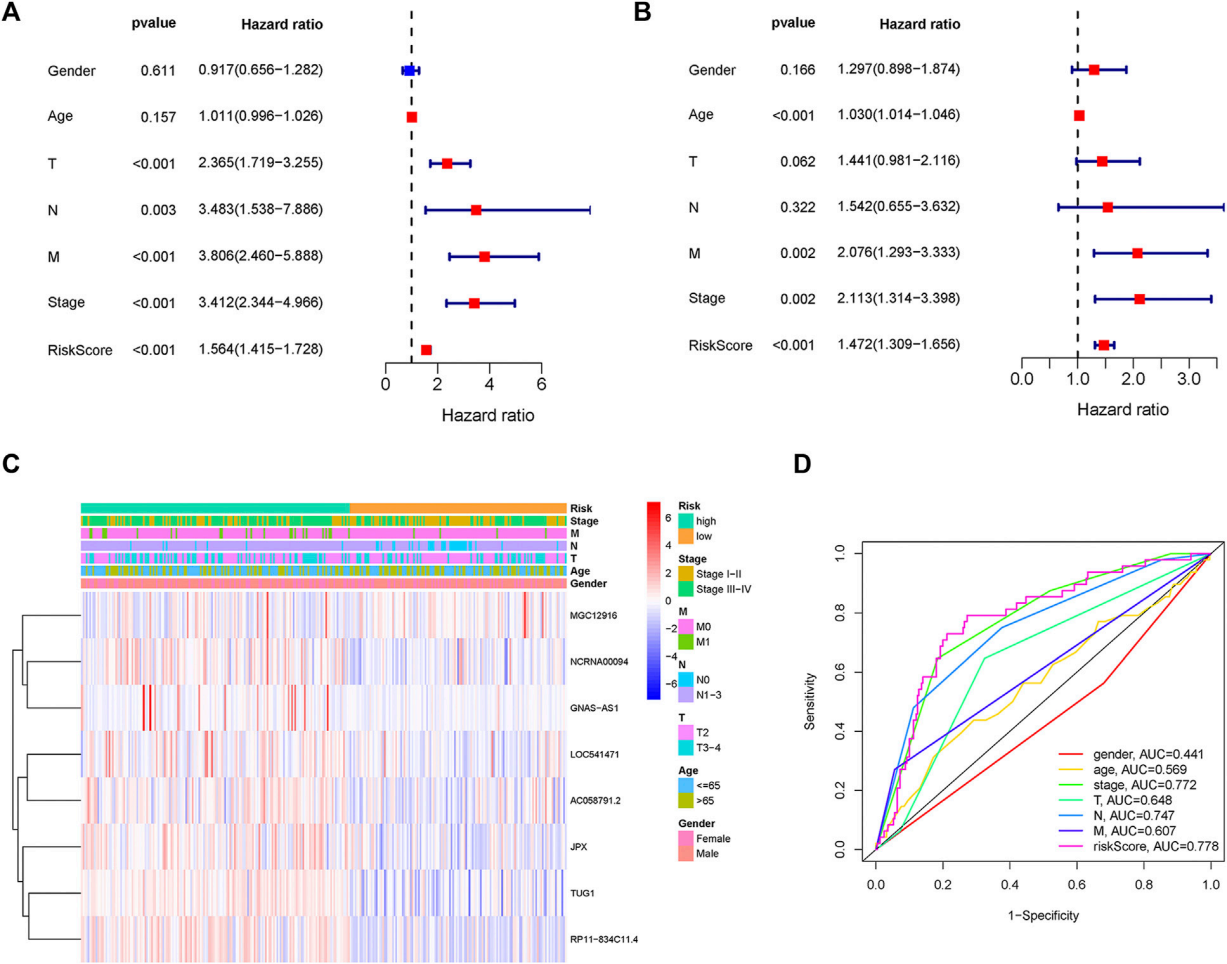
PRLs risk signature identified as an independent prognostic factor. **(A)** Univariate analysis displayed that PRLs prognostic risk scores (*p* < 0.001) and other clinical characteristics including T, N, M and stage were markedly associated with survival time. **(B)** Multivariate analysis indicated that prognostic risk score (*p* < 0.001), age, T, M and as well as stage were independent prognostic factors. **(C)** Heat map displayed the expression level of the eight PRLs in the risk model and the clinicopathological characteristics of patients with gastric cancer. **(D)** Comparison of ROC curves analysis indicated the AUC values among the risk score and clinicopathological features.

**FIGURE 7:**
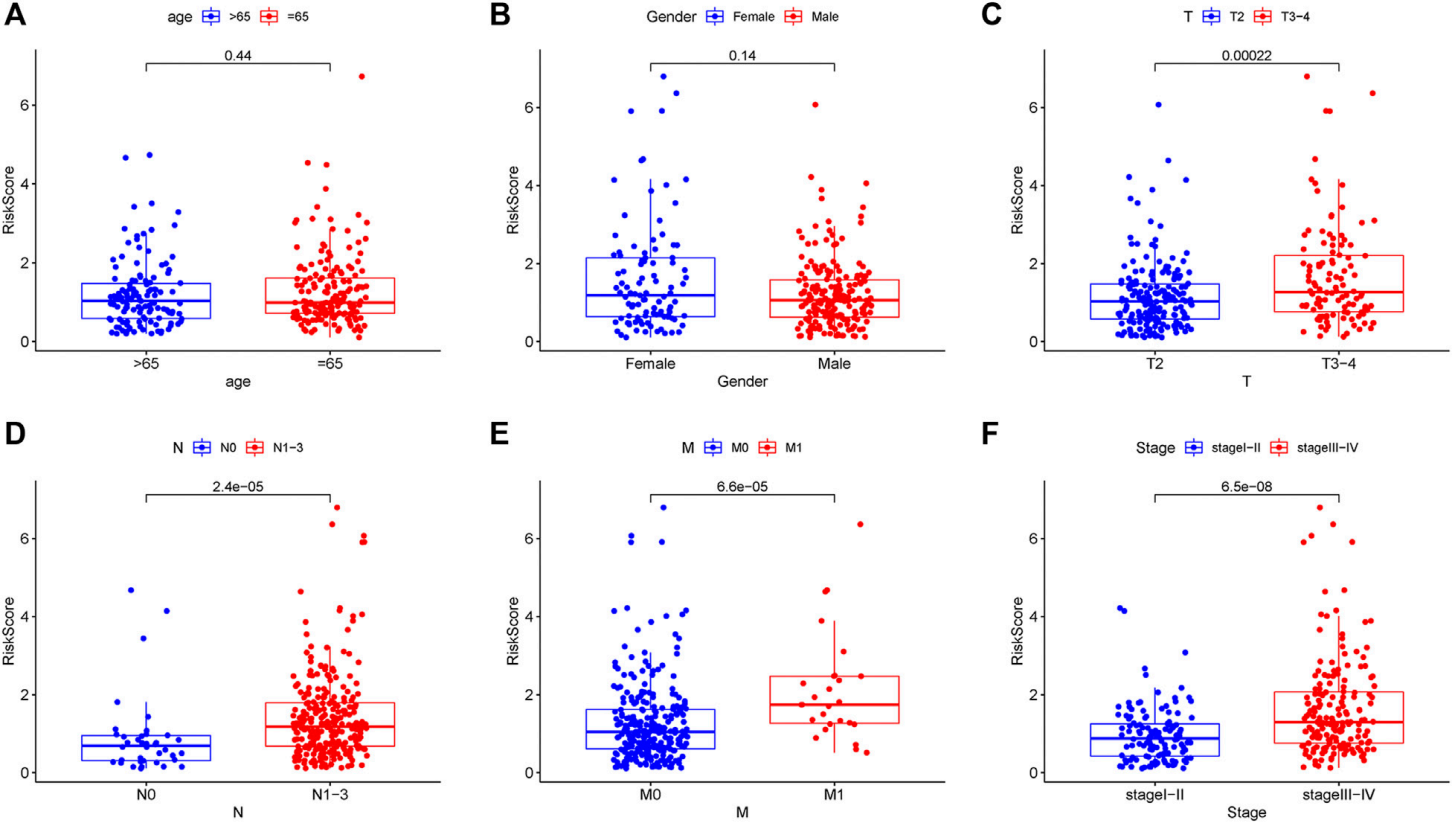
Correlation analysis between prognostic PRLs risk score and different clinical features. The PRLs risk score showed no difference in **(A)** age (*p* = 0.44) and **(B)** gender (*p* = 0.14). The PRLs risk score displayed significant difference in **(C)** T (*p* < 0.001), **(D)** N (*p* < 0.001), **(E)** M (*p* < 0.001) and **(F)** stage (*p* < 0.001).

**FIGURE 8:**
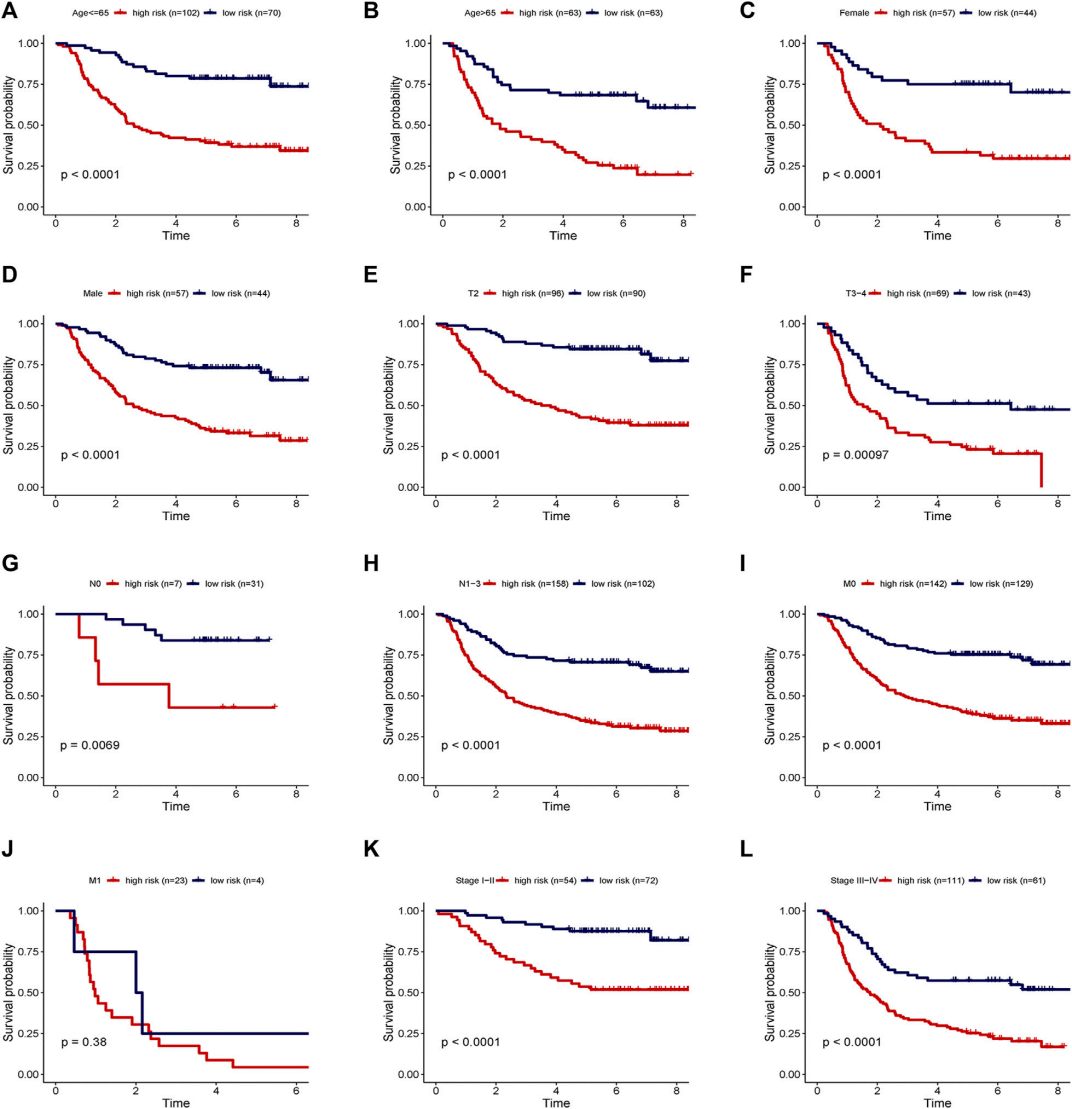
Stratification analyses of survival curve. Survival analysis showed the high-risk group had worse survival than the low-risk group, when stratified by **(A,B)** age, **(C,D)** gender, **(E,F)** T, **(G,H)** N, **(I,J)** M and **(K,L)** stage.

Two patients lacked a detailed clinical index in GSE62254 were removed, the lncRNA expression and clinical information of the remaining 298 patients were retained (Supplementary Datasheet S4). To assess the independence of the risk signature model, univariate and multivariate analysis were carried out on account of integrated the risk signature with other clinical factors including gender, age, T, N, M, and stage. Interestingly, the uni- and multivariate analysis both demonstrated that PRGs risk signature could predict the prognosis independently in patients of GC (*p* < 0.001 and *p* < 0.001, respectively) (Figures 6A,B). The heatmap showed that the expression of eight PRLs signature was also associated with the clinicopathological features of GC (Figure 6C). An AUC value of 0.778 based on the prognostic model was markedly better than the value of clinical indicators including gender (0.441), age (0.569), stage (0.772), T (0.648), N (0.747) and M (0.607) (Figure 6D). Although the risk score of the PRL signature showed no difference in age and gender, it was statistically significant higher in high T, early lymph node metastasis, more prone to distal metastasis and advanced stage (Figure 7). Moreover, as shown in Figure 8, the stratified analysis based on clinicopathological features demonstrated that the GC patients in the low-risk group had better OS in different stratums, such as age (>65 years or≤65 years), gender (female or male), clinical T (T2 or T3-4), clinical N (N0 or N1-3), clinical M (M0 or M1) and stage (stage III-IV or stage I-II). The results pointed that the PRLs signature was still remained powerful to predict GC survival in different gradation of age, gender, T, N, M and stage.

### Construction and evaluation of nomogram

**FIGURE 9:**
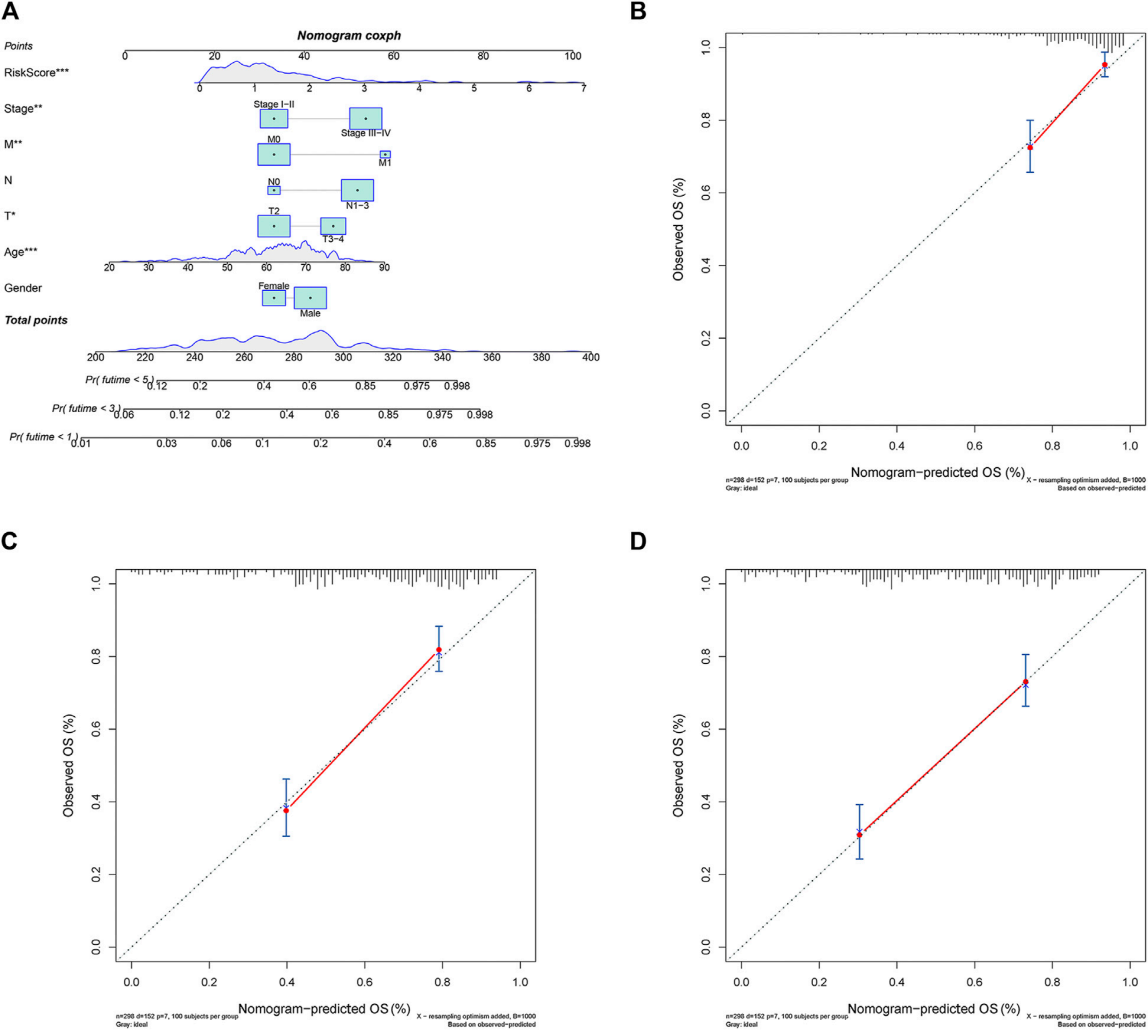
The 1, 3, 5-year survival probability of patients with gastric cancer were predicted through nomogram. **(A)** Prognostic nomogram of gastric cancer patients; Calibration curves for the nomogram at **(B)** 1-, **(C)** 3-, and **(D)** 5-year.

A nomogram predicting GC patients’ 1-, 3- and 5-year survival probability was constructed based on the comprehensive landscape of the integrated patients’ risk scores and clinical features. As shown in Figure 9A, seven prognostic parameters were selected into the nomogram, including the PRLs risk signature and gender, age, T, N, M, as well as stage. In terms of the 1-, 3- and 5-year survival rates, a high level of consistency was displayed in calibration plots between actual observations and nomogram predictions (Figures 9B–D).

### Comparison of immunological activity between subgroups

**FIGURE 10:**
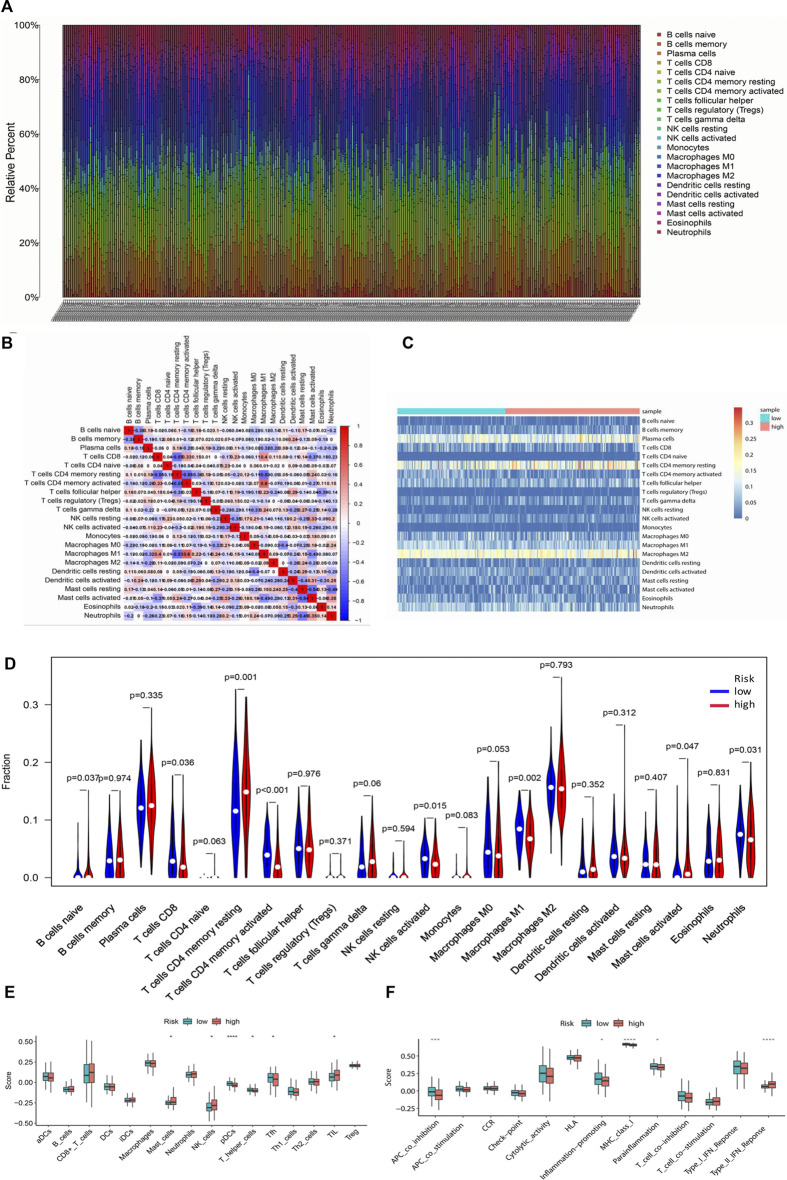
Tumor-infiltrating immune cells between the low-risk and high-risk groups. **(A)** The proportions of different tumor-infiltrating immune cells in individual gastric cancer patients. **(B)** Heat map showed the correlation between the 22 kinds of immune cells. **(C)** Heat map of immune cell abundance of the 22 kinds of immune cells in the low-risk group and high-risk group. **(D)** The different proportions of the 22 kinds of immune cells between the high-risk group and low-risk group were demonstrated by violin plot. **(E)** Boxplots showed the different enrichment scores of 16 types of immune cells in the high-risk group and low-risk group. **(F)** Boxplots showed the different enrichment scores of 13 immune-related pathways in the high-risk group and low-risk group.

CIBERSORT was implemented to evaluate the relationship between the risk score and the 22 types of TIICs in GC (Figures 10A,C). Among the immune cells, Pearson correlation analysis was performed (Figure 10B). After further analysis, it was illustrated that there were higher proportions of B cells naïve and T cell CD4 memory resting, while lower proportions of T cells CD8, T cell CD4 memory activated, NK cells activated, Macrophages M1, and Neutrophils in the high-risk group (Figure 10D). Then the enrichment scores of 16 types of immune cells and 13 immune-related pathways were studied in the GSE62254 dataset by using ssGSEA. Intriguingly, in the high-risk group, immune cells such as Mast_cells, NK_cells, and TIL had significantly higher enrichment scores, while pDCs, T_helper_cells and Tfh had significantly lower enrichment scores (Figure 10E). In addition, the high-risk group also had markedly lower scores on four immune pathways, including APC_co_inhibition, Inflammation-promoting, MHC_class_1 and Parainflammation, while Type_II_IFN_Response was much higher in high-risk group (Figure 10F). These results indicated that the differentially displayed immune cells in the two groups might have an important impact on the prognosis of GC patients.

## Discussion

In our study, a multistep re‐annotation analysis of lncRNA and pyroptosis gene expression was performed in gastric cancer. Based on bioinformatics analysis, their expression profiles in GSE62254 of gastric cancer were analyzed. One group of lncRNA expression level was found to be related with pyroptosis mRNA expression. Intriguing, the pyroptosis-related lncRNAs (PRLs) signature with prognostic value was established *via* Cox proportional hazard and LASSO models. Its robustness was also well validated in the GSE62254 internal validation set and the GSE57303 external validation set. Moreover, nomogram for predicting the 5-year survival probability of patients with GC was constructed. Further, the relationship between immune cell infiltration/pathway and risk group was explored.

Gastric cancer with an incidence rate of fifth and the mortality rate of fourth globally caused a heavy burden in most countries. Pyroptosis know as a new form of programmed cell death, play a dual-role in cancer development and treatment [21]. First, the normal cells are stimulated by many inflammatory factors released from thermal prolapse, result in transforming into tumor cells. Meanwhile, a new therapeutic target may come from the promotion of cancer cell pyroptosis [22, 23]. Nowadays, the emergence of thousands of lncRNAs were demonstrated to participate in cellular mechanisms widely, from almost all aspects of gene expression to protein translation and stability [25, 26]. Therefore, whether some lncRNAs are associated with pyroptosis in gastric cancer sample needs in deep study. To demonstrate it, a multistep re‐annotation analysis of lncRNA and pyroptosis gene expression was performed in gastric cancer from profiles in GSE62254. Fortunately, some lncRNAs expression level was found to be related with pyroptosis messenger RNA (mRNA) expression (*p* < 0.001), named as pyroptosis-related lncRNAs. Interestingly, these lncRNAs were studied in different diseases separately. JPX regulated GC development through miR-197/CXCR6 axis [27] and yet related with non-small cell lung cancer [28] and cervical cancer [29]. GNAS-AS1 promoted migration and invasion of in nasopharyngeal carcinoma [30], non-small cell lung cancer [31] and etc. AC058791.2 was associated with prognosis of breast cancer [32] and therapy-resistant asthma [33]. RP11−834C11.4 together with other lncRNAs acted as risk factors in colon cancer [34, 35]. TUG1 played important role in the development or progression of hepatocellular carcinoma [36], stomach adenocarcinoma [37] and Bladder Cancer [38]. MGC12916, NCRNA00094 and LOC541771 have been rarely studied. However, our study was the first one to recognize the eight lncRNAs as PRLs and combined them together in gastric cancer.

In the past decades, a variety of literature have been reported that the pyroptosis promotes the development and/or progression of human cancers. In Wang’s study, it has been illustrated that pyroptosis facilitates esophageal cancer progression *via* alcohol accumation-induced esophagitis [39]. Chu et al. demonstrated the potential role of pyroptosis-mediated cell death in the development and progression of hepatocellular carcinoma [40]. Currently, Wang and others reported that GSDME promotes gastric cancer progression through caspase-3-dependent pyroptosis [41]. Moreover, functional lncRNAs expression patterns were associated with different human cancers [42, 43]. The deregulation of some lncRNAs expression is associated with cancer progression and patient prognosis. Actually, a survey of lncRNA expression patterns among different cancer types has implied that the abnormal expression of lnRNA may display a better specificity to cancer type and grade compared to alterations in the expression of messenger RNAs [44–46]. These correlative findings have provided initial indications that lncRNAs may represent an unexplored reservoir of diagnostic and prognostic markers in cancer [47]. However, little literature reported the function of PRLs signature in the gastric cancer [24]. As the poor prognosis of gastric cancer, it is still necessary to select appropriate therapeutic method for cancer patients *via* prognostic assessment. In light of that, the PRLs signature with prognostic value was established *via* Cox proportional hazard and LASSO models. Patients with low-risk score had better overall survival than those with high-risk score notably (Log-rank test, *p* < 0.0001). Moreover, the PRLs signature could predict the prognosis in gastric cancer independently. Consequential, its robustness was also well validated in the GSE62254 internal validation set and the GSE57303 external validation set. Nomogram for predicting the 5-year survival probability of patients with GC was constructed.

Pyroptosis has a close relationship with inflammation and tumor immunity [48]. However, topics focusing on the role of PRLs in immune microenvironment of tumor is rare. Thus, we investigate the relationship between immune cell infiltration/pathway and risk group. Intriguingly, the high-risk group observed lower proportion of T cells CD8, T cells CD4 memory activated, NK cells activated, macrophages M1, and neutrophils. Moreover, the high-risk group also observed decreased immune score of APC_co_inhibition, Inflammation-promoting, MHC_class_1 and Parainflammation. Both proportion and immune score of immune cells infiltration assay displayed that T cells CD4, NK cells, Macrophages and Neutrophils were associated with the two risk groups. In line with this, Chen and his colleagues reported that GC patients with higher immune score and higher abundance of CD8^+^ T cells, CD4^+^ T–activated cells, follicular helper T cells, M1 macrophages, and NK cells had better prognosis [49]. This is the first time to demonstrate that higher level of the eight PRLs had lower immune cells infiltration of CD4^+^ T–activated cells, M1 macrophages, Neutrophils and NK cells. Again, the high-risk group exhibited suppression in several immune pathways, such as APC_co_inhibition, Inflammation-promoting, MHC_class_1 and Parainflammation. In one word, our results implied that inflammation and tumor immunity associated with pyroptosis may involve in GC prognosis.

Despite the novel PRLs signature found in our study was robust, several limitations were still existed. On the one hand, a large quantity of clinical sample of gastric cancer was need to verify the PRLs signature further. On the other hand, we had better verify those lncRNAs, immune cells and immune checkpoint proteins in GC by cellular experiment.

In conclusion, we established that the PRLs signature was associated with prognostic value through Cox proportional hazard and LASSO models. The robustness of the PRLs signature was also validated in internal validation set and external validation set. Then, the prognosis prediction model of gastric cancer was constructed in nomograms based on lasso-regression analysis. Finally, immune cell enrichment analysis demonstrated that inflammation and tumor immunity associated with pyroptosis may involve in GC development. Therefore, the PRLs signature may be a potential biomarker to evaluate the prognosis of patients with gastric cancer, and these lncRNAs may play a key role in the development and treatment of gastric cancer trough immune pathway.

## Data Availability

The original contributions presented in the study are included in the article/Supplementary Material, further inquiries can be directed to the corresponding author.
